# Hyaluronic Acid as an Adjunct in Bone Regeneration—A Systematic Review

**DOI:** 10.3390/biomedicines14071514

**Published:** 2026-07-05

**Authors:** Lola Hennebelle, Cátia Reis, Marta Relvas, Filomena Salazar, Rosana Costa, Cristina Cabral, Ana Sofia Vinhas

**Affiliations:** 1 Faculty of Dental Medicine, University Institute of Health Sciences (IUCS), Cooperativa de Ensino Superior Politécnico e Universitário (CESPU), 4585-116 Gandra, Portugal; a31891@alunos.cespu.pt; 2 Department of Medicine and Oral Surgery, University Institute of Health Sciences (IUCS-CESPU), 4585-116 Gandra, Portugal; catia.reis@iucs.cespu.pt (C.R.); marta.relvas@iucs.cespu.pt (M.R.); filomena.salazar@cespu.pt (F.S.); rosana.costa@iucs.cespu.pt (R.C.); cristina.cabral@iucs.cespu.pt (C.C.); 3 Oral Pathology and Rehabilitation Research Unit (UNIPRO), University Institute of Health Sciences (IUCS-CESPU), 4585-116 Gandra, Portugal

**Keywords:** hyaluronic acid, bone regeneration, maxillary sinus, oral surgical procedures, tooth extraction

## Abstract

**Background:** Bone tissue is a dynamic structure capable of continuous remodeling; however, its regenerative capacity is limited in critical-size defects, often requiring the use of bone grafting procedures. Available grafting materials present inherent limitations, highlighting the need for strategies that can enhance regenerative outcomes. Hyaluronic acid (HA) has been proposed as a promising adjunctive agent because of its biological properties, including anti-inflammatory and pro-angiogenic effects. **Objective:** To systematically evaluate the available clinical evidence regarding the effects of HA as an adjunct in bone regeneration procedures, including alveolar ridge preservation, ridge augmentation, and maxillary sinus elevation. **Materials and Methods:** A systematic search was conducted in the PubMed, ScienceDirect, Google Scholar, and Wiley Online Library databases for studies published within the last 10 years. Clinical studies involving adult patients were included if they evaluated the local application of HA, regardless of formulation, and reported quantitative clinical, radiographic, histological, or histomorphometric outcomes related to bone regeneration. **Results:** Of the 728 records initially identified, 10 studies met the eligibility criteria and were included in the qualitative synthesis. **Discussion:** Overall, the available evidence suggests that HA may positively influence bone regeneration outcomes. The most consistent benefits were observed in alveolar ridge preservation and ridge augmentation procedures, including increased new bone formation, improved bone density, enhanced bone maturation, and reduced dimensional bone loss. In contrast, findings regarding maxillary sinus augmentation were less consistent. **Conclusions:** HA appears to be a promising adjunct in bone regeneration procedures. However, the current evidence remains limited and is primarily based on clinical outcomes, providing insufficient mechanistic data to fully elucidate its biological effects. Further well-designed randomized controlled trials with standardized protocols are required before definitive clinical recommendations can be established.

## 1. Introduction

Bone tissue is far more than a simple rigid structure; it is a living, dynamic, and highly organized tissue that undergoes continuous renewal through a process known as bone remodelling [1]. However, this regenerative capacity has limitations, particularly in cases of significant bone loss. When a defect reaches a critical size, spontaneous regeneration becomes insufficient, often requiring surgical intervention. In such situations, bone grafting represents one of the most used treatment approaches, aiming to promote effective and stable healing while restoring the lost anatomical structure [2,3].

Alveolar bone is a tooth-dependent tissue that undergoes progressive resorption following tooth loss. In implant dentistry, grafting procedures are widely used to maintain or restore adequate bone volume and density, which are essential for implant stability and successful osseointegration. Insufficient bone volume may compromise the predictability and long-term success of implant-supported rehabilitation [2,3]. Previous systematic reviews have demonstrated the benefits of alveolar ridge preservation procedures in reducing post-extraction dimensional changes and improving conditions for subsequent implant placement [4,5].

Accordingly, bone grafts are frequently used in regenerative procedures such as alveolar ridge augmentation and maxillary sinus elevation to recreate favorable anatomical conditions for oral rehabilitation. Following tooth extraction, grafting materials may also be used for socket preservation, helping to maintain alveolar dimensions and facilitate future interventions. In periodontal therapy, bone grafts play an important role in the regeneration of supporting tissues, contributing to tooth stability and reducing the progression of bone loss [3]. The clinical effectiveness of different grafting materials in ridge augmentation and maxillary sinus augmentation has been extensively investigated, highlighting both their regenerative potential and inherent limitations [6,7].

To achieve these clinical objectives, different types of grafting materials may be used, each with specific advantages and limitations. Autogenous grafts, in which bone is harvested from the patient, remain the gold standard due to their osteogenic, osteoinductive, and osteoconductive properties. However, their use is associated with several drawbacks, including donor-site morbidity, postoperative pain, risk of infection, healing complications, and limited availability of graft material, particularly in extensive reconstructive procedures [3].

In contrast, allogeneic grafts, obtained from human donors, eliminate donor-site morbidity but may be associated with immunological risks and variability in osteoinductive potential. Xenogeneic grafts, derived from animal sources, are widely used in dentistry because of their excellent osteoconductive properties; nevertheless, ethical concerns and a minimal risk of disease transmission remain relevant considerations. Synthetic biomaterials also represent widely used alternatives and include materials such as beta-tricalcium phosphate (β-TCP), hydroxyapatite (HAp), biphasic calcium phosphate (BCP), and bioactive glasses. These biomaterials are characterized by high biocompatibility, osteoconductive properties, ease of handling, and a very low risk of disease transmission. However, because they generally lack intrinsic osteogenic and osteoinductive properties, their regenerative potential may be limited when compared with autogenous grafts [3,8,9].

Given these limitations, there is a growing need to develop strategies capable of enhancing bone regeneration and optimizing the clinical outcomes of grafting procedures, particularly through the incorporation of bioactive agents [2,3].

In this context, hyaluronic acid (HA) has attracted increasing interest in dentistry. Naturally present in the human body, this glycosaminoglycan is an essential component of the extracellular matrix and plays a crucial role in tissue hydration, cell–matrix interactions, and wound healing. In addition, HA exhibits anti-inflammatory and antibacterial properties, supporting its potential application in regenerative therapies [10,11,12].

Within bone tissue, HA interacts with cells involved in bone metabolism, including osteoblasts, osteoclasts, and fibroblasts, as well as extracellular matrix components such as collagen and growth factors. These interactions are believed to support biological processes including cell migration, proliferation, and differentiation, which may contribute to bone regeneration [10,11,12].

Widely used in medical fields such as ophthalmology, dermatology, and orthopedics [12], HA has also gained increasing relevance in dentistry, particularly in oral surgery. Its potential use as an adjunctive biomaterial in bone grafting procedures represents a promising strategy for improving regenerative and clinical outcomes [10,11].

Despite a growing body of experimental and clinical research, the available evidence remains heterogeneous and, in some cases, contradictory, owing to differences in study design, defect characteristics, biomaterials, and HA formulations [10,11]. Recent evidence has reinforced the regenerative potential of HA in bone healing. For example, a systematic review by Surroca et al. reported that HA may accelerate bone repair through modulation of inflammatory responses, stimulation of angiogenesis, and enhancement of osteogenic activity [13]. Although that review focused primarily on fracture healing rather than oral bone regeneration, its findings support the biological rationale for the use of HA as an adjunctive regenerative agent and highlight the need for further investigation in dental applications.

Hyaluronic acid has been investigated as a potential adjunctive agent in bone regeneration because experimental and preclinical studies have suggested possible effects on biological processes involved in tissue repair, including inflammation modulation, angiogenesis, and cellular activity. However, the extent to which these mechanisms translate into clinically meaningful regenerative outcomes remains unclear. Consequently, it remains uncertain whether the proposed biological effects translate into clinically meaningful benefits.

Given the diversity of the available evidence and the limited understanding of the mechanisms underlying the observed clinical outcomes, a systematic review is warranted to critically synthesize the existing literature. Therefore, the aim of this systematic review was to evaluate the available clinical evidence regarding the effects of HA as an adjunct in bone grafting procedures, particularly in post-extraction socket preservation, alveolar ridge augmentation, and maxillary sinus elevation.

## 2. Materials and Methods

### 2.1. Study Protocol

The eligibility criteria for the included studies were established based on the PICO framework (Population, Intervention, Comparison, and Outcomes) as presented in Table 1. This systematic review was conducted in accordance with the PRISMA 2020 guidelines [14]. The review protocol was previously registered in the PROSPERO database under registration number CRD420261384659.

PICO question. The following focused question was formulated according to the study design, population, intervention, comparison, and outcomes: In adult patients undergoing socket preservation, alveolar ridge augmentation, or maxillary sinus elevation procedures (P), does the adjunctive use of hyaluronic acid (I), compared with conventional techniques without hyaluronic acid (C), improve bone regenerative outcomes, namely bone volume gain, width and height, percentage of new bone formation, bone density, and bone maturation (O)?

### 2.2. Search Strategy

A literature search was conducted between January 2016 and January 2026 in PubMed, ScienceDirect, Google Scholar, and Wiley Online Library databases using the following keywords: “hyaluronic acid,” “bone regeneration,” “maxillary sinus,” “oral surgery procedures,” and “tooth extraction.” An additional manual search was conducted in PubMed using the terms: “hyaluronic acid,” “bone remodeling,” “bone graft,” “dentistry,” and “bone autoregeneration.”

#### 2.2.1. Search Terms

An advanced database search was performed in PubMed, ScienceDirect, Google Scholar, and Wiley Online Library using the following combinations of MeSH terms, with a 10-year publication filter:

- (“hyaluronic acid”[MeSH Terms]) AND (“oral surgical procedures”[MeSH Terms])
- “hyaluronic acid” AND “maxillary sinus”
- “hyaluronic acid” AND “bone regeneration” AND “tooth extraction” (excluding animals and reviews)
- “hyaluronic acid” AND “bone regeneration” AND “maxillary sinus”

#### 2.2.2. Inclusion and Exclusion Criteria

This systematic review included clinical studies involving adults aged 18 years or older who underwent socket preservation, alveolar ridge augmentation, or maxillary sinus elevation procedures. Eligible studies evaluated local application of hyaluronic acid (HA), regardless of formulation, including gel forms, cross-linked HA, or combinations with biomaterials such as bone grafts. Only full-text articles published between January 2016 and January 2026 in English, Portuguese, or Spanish, were considered. To be eligible, studies were required to report quantitative clinical, radiographic, histological, or histomorphometric outcomes related to bone preservation or regeneration. Furthermore, only studies with an appropriate control group that allowed comparison between procedures performed with and without the use of HA were selected. These criteria are summarized in Table 2.

In vitro studies, animal studies, computational simulations, or any other non-clinical experimental designs were excluded. Case reports, case series without a control group, conference abstracts, and narrative reviews were also excluded. Studies involving systemic use of HA, whether oral or injectable, were not considered, as only its local application, during regenerative procedures, was relevant to the objectives of this review. Finally, studies lacking an appropriate comparative control group were excluded from the analysis.

#### 2.2.3. Study Selection

The following filters were applied, when available, in the selected databases: publication period (January 2016–January 2026), language (English, Portuguese, or Spanish), and human studies. Eligibility criteria regarding participant age (≥18 years), study design, intervention, and outcome measures were subsequently applied during the screening and full-text assessment phases. After the predefined search filters had been applied, duplicate records were removed. Title and abstract screening were then performed independently by two reviewers according to the predefined inclusion and exclusion criteria. Studies considered potentially eligible by either reviewer were selected for full-text assessment. Any disagreements regarding study eligibility were resolved through discussion and consensus, and when necessary, a third reviewer was consulted. Manuscripts that did not meet the eligibility criteria during the screening phase were excluded. The remaining potentially relevant studies were retrieved and assessed in full text by the same reviewers. Only studies fulfilling all inclusion criteria were included in the final qualitative synthesis of this systematic review.

### 2.3. Data Extraction

A standardized data extraction form was developed to collect relevant information from the studies included. Data extraction was performed independently by two reviewers. The following information was recorded for each study: authors, year of publication, study design, clinical indication (alveolar ridge preservation, ridge augmentation, or maxillary sinus elevation), sample size, intervention and control groups, follow-up period, outcome measures, and main findings. Any discrepancies between reviewers were resolved through discussion and consensus.

### 2.4. Methodological Quality Assessment

Risk of bias assessment was performed independently by two reviewers, using validated tools appropriate to the methodological design of each study, in accordance with PRISMA 2020 recommendations. Any disagreements between reviewers were resolved by consensus.

## 3. Results

### 3.1. Search Results

The literature search was conducted using keywords and MeSH terms related to the topic of interest. The search strategies are detailed in Table 3.

**Figure 1:**
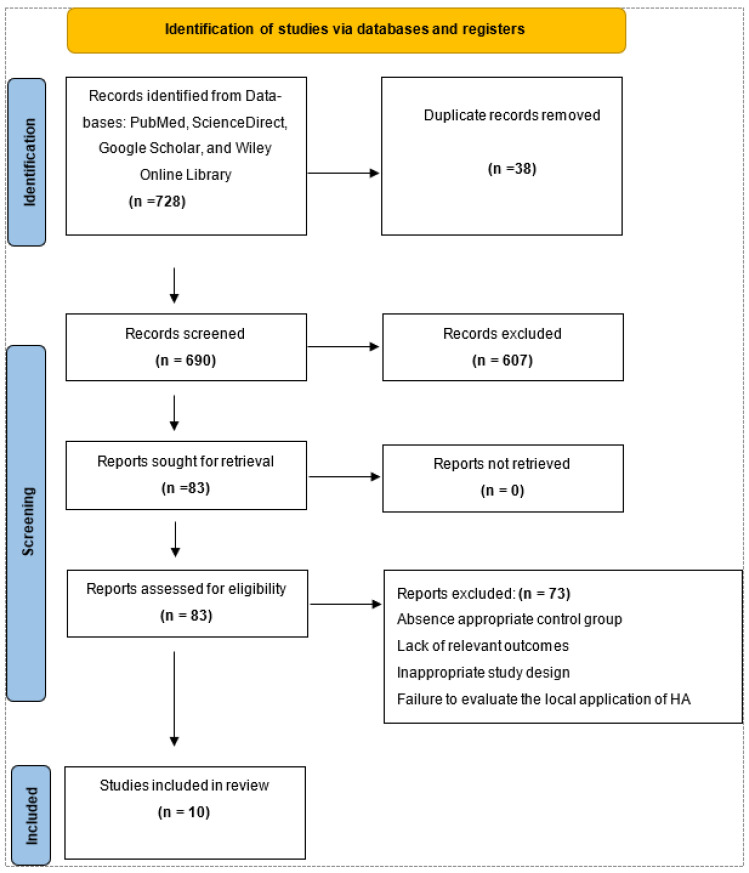
Flowchart of the Search Strategy (PRISMA).

The initial search identified 728 records from PubMed, ScienceDirect, Google Scholar, and Wiley Online Library databases. After the removal of 38 duplicate records, 690 records remained for title and abstract screening. During the screening phase, 607 records were excluded for not meeting the predefined inclusion criteria. The remaining 83 articles were considered potentially eligible and were retrieved for full-text assessment. Following full-text evaluation, 73 studies were excluded due to the absence of an appropriate control group, lack of relevant quantitative clinical, radiographic, histological, or histomorphometric outcomes, inappropriate study design (e.g., animal studies, in vitro studies, reviews, or case reports), or failure to evaluate the local application of hyaluronic acid in bone regeneration procedures. Consequently, 10 studies met all eligibility criteria and were included in the qualitative synthesis. The study selection process is illustrated in the PRISMA flow diagram (Figure 1).

### 3.2. Characteristics of the Studies

Ten clinical studies were included, comprising nine randomized clinical trials (RCTs), four of which employed a split-mouth design) and one comparative clinical study. Most studies used cross-linked hyaluronic acid formulations, particularly Hyadent BG^®^. Considerable heterogeneity was observed regarding the formulation, concentration, and molecular characteristics of hyaluronic acid, including molecular weight and degree of cross-linking. A summary of these characteristics is presented in Table 4.

### 3.3. Methodological Assessment of the Studies

The methodological assessment of the included studies, namely randomized controlled trials (RCTs) and comparative clinical studies, was conducted to evaluate scientific quality, internal validity, and risk of bias. Overall, methodological heterogeneity was observed among the studies, as well as limitations related to small sample sizes and lack of blinding in some cases, which may compromise the robustness and comparability of the results. Table 5 summarizes the methodological evaluation of the studies included by assessment tool.

#### 3.3.1. Randomized Controlled Trials (RCTs)

For randomized controlled trials, methodological quality was assessed using the Cochrane Risk of Bias 2.0 (RoB 2) tool. This instrument provides a structured, domain-specific evaluation of risk of bias across five key domains: (1) bias arising from the randomization process; (2) bias due to deviations from intended interventions; (3) bias due to missing outcome data; (4) bias in outcome measurement; and (5) bias in selection of the reported result. Each domain was rated as “low risk of bias,” “some concerns,” or “high risk of bias,” leading to an overall judgment for each study. The most common sources of bias among the randomized controlled trials were, insufficient reporting of allocation concealment, lack of protocol registration, and limited information regarding selective outcome reporting, resulting in an overall judgment of “some concerns” for most studies. Three studies (Helal et al., 2025; Velasco-Ortega et al., 2020; and Dogan et al., 2017 [21,23,24]) were classified as low risk of bias across all RoB 2 domains.

#### 3.3.2. Non-Randomized Intervention Studies

Comparative and non-randomized prospective clinical studies were assessed using the ROBINS-I (Risk Of Bias In Non-randomized Studies of Interventions) tool. This instrument evaluates risk of bias across seven domains, including bias due to confounding, selection of participants, classification of interventions, deviations from intended interventions, missing data, measurement of outcomes, and selection of reported results. The overall risk of bias is categorized as “low,” “moderate,” “serious,” or “critical.” The non-randomized comparative study by Kloss et al. (2024) [16] was judged as having a moderate risk of bias, mainly due to potential confounding and participant selection bias inherent to its non-randomized design.

Detailed domain-specific risk-of-bias assessments and summary traffic-light plots of the RoB 2 and ROBINS-I assessments are presented in Supplementary Materials (Tables S1 and S2 and Figures S1 and S2).

### 3.4. Results Table

Results table was stratified according to the surgical procedure evaluated (alveolar ridge preservation, alveolar ridge augmentation, and maxillary sinus elevation) and, within each category, studies were arranged in descending order according to year of publication, from the most recent to the oldest, as shown in Table 6.

## 4. Discussion

This systematic review aimed to critically analyze the available scientific evidence regarding the role of hyaluronic acid (HA) as an adjuvant in bone regeneration procedures, including alveolar ridge preservation (socket and ridge preservation), ridge augmentation, and maxillary sinus elevation. Ten clinical studies involving 193 participants, of which six addressed alveolar ridge preservation [15,16,17,18,19,20], two evaluated ridge augmentation procedures [21,22], and two assessed maxillary sinus elevation [23,24].

Most studies were randomized controlled trials (RCTs) [15,17,18,19,20,21,22,23,24], including several split-mouth designs [17,20,21,24], which strengthens the quality of evidence by reducing bias and inter-individual variability. However, the inclusion of one comparative clinical study [16], together with the methodological heterogeneity observed across studies, represents an important limitation that may affect the comparability and generalizability of the findings. Despite these limitations, the available evidence suggests that HA may positively influence bone regeneration outcomes, although the overall strength of the evidence remains limited.

For clarity, the discussion is organized according to the regenerative procedures evaluated: alveolar ridge preservation, ridge augmentation, and maxillary sinus elevation.

Regarding alveolar ridge preservation, the results overall suggest that HA has a beneficial effect on post-extraction bone regeneration.

From a radiographic perspective, several included studies reported significantly greater bone density in HA-treated groups. For example, Abdelzaher et al. and Baiomy et al. observed higher density values in the test groups compared with controls [18,19], a finding also reported by Kloss et al., who demonstrated a progressive improvement in grey values after 4 months of healing [16]. Similarly, Taman et al. found a significant short-term increase in bone density, particularly after 2 months [20]. This enhancement may reflect accelerated mineralization and bone maturation processes, suggesting a more favorable environment for development of organized and dense bone formation during healing.

The effects of HA on post-extraction bone resorption appear to be more variable and may depend on the specific dimensional parameter evaluated (vertical, volumetric, or horizontal). Several studies reported significantly lower vertical bone loss in the HA groups compared with the controls [15,16,19]. Likewise, most investigations evaluating volumetric changes suggested a beneficial effect of HA on bone volume preservation [16,17,19]. This observation is further supported by linear measurements, which consistently demonstrated reduced dimensional loss in the treated groups [17].

However, results regarding horizontal dimensions are more variable. While one study [15] demonstrated a beneficial effect of HA on alveolar width preservation, others [16,19] found no statistically significant differences between groups.

This suggests that HA may have a more consistent effect on maintaining bone volume and height, while its influence on horizontal dimensions remains less predictable.

Overall, the radiographic evidence suggests that HA may contribute to increased bone density, improved dimensional stability, and enhanced preservation of bone volume.

Clinically, HA does not appear to exert a significant effect on horizontal bone width, according to Abaza et al. [15]. In addition, postoperative pain was evaluated in several studies [18,19,20], with no significant differences observed between treatment and control groups. Taman et al. also assessed implant stability and reported no statistically significant differences associated with HA use [20].

Histological and histomorphometric findings indicate that HA is associated with a greater amount of newly formed bone [15,17]. Furthermore, improvements in bone maturation were reported by Abaza et al., Husseini et al., Baiomy et al., and Taman et al. [15,17,19,20], suggesting a positive influence of HA on the quality and organization of the regenerated tissue.

When observed, a lower amount of residual biomaterial, may reflect a more advanced bone remodelling process. Husseini et al. and Baiomy et al. reported findings consistent with a favorable influence of HA on this parameter [17,19], whereas Abaza et al. did not observe statistically significant differences in the amount of residual graft material [15].

Taken together, the available evidence from alveolar ridge preservation studies indicates that HA may promote osteogenesis and enhance the quality and stability of regenerated tissues.

Evidence regarding ridge augmentation remains limited, as conclusions are based on only two randomized controlled trials [21,22]. Consequently, the available findings should be interpreted with caution.

With respect to bone gain, Helal et al. reported significantly improved outcomes in the HA-treated group, suggesting a beneficial effect on bone formation and dimensional graft stability during the healing period [21].

Clinically, HA may contribute to reduce crestal width loss, as reported by Kauffmann et al. [22]. These findings contrast with those of Abaza et al. [15], who found no statistically significant effect of HA on alveolar ridge dimensions following socket preservation.

Histological and histomorphometric analyses further support a beneficial role of HA in ridge augmentation procedures. Helal et al. and Kauffmann et al. reported significantly greater amounts of newly formed bone in the HA-treated groups compared with the controls [21,22]. Moreover, Kauffmann et al. observed a higher degree of mineralization and a lower proportion of residual biomaterial, whereas Helal et al. reported enhanced bone maturation [21,22]. Collectively, these findings suggest that HA may promote a more efficient bone remodeling process, like that observed in alveolar ridge preservation procedures.

Finally, studies evaluating maxillary sinus elevation were examined. The available evidence remains limited and somewhat heterogeneous.

Dogan et al. reported that the addition of HA to graft biomaterials enhanced new bone formation during maxillary sinus augmentation [24]. A significant increase in newly formed bone was observed when an HA matrix was combined with a collagenated xenograft, as demonstrated by both micro-CT and histomorphometric analysis. The authors also reported evidence of enhanced neovascularization. These observations may be related to the biological properties of HA, particularly its ability to improve clot stability and stimulate angiogenesis, both of which are essential in the relatively poorly vascularized environment of the maxillary sinus [24].

However, the available evidence remains inconsistent across studies and follow-up periods. Velasco-Ortega et al. evaluated different biomaterials used in maxillary sinus augmentation, including ABBM and TCP with or without HA addition, and found no significant differences between groups, after 9 months in terms of bone formation, maturation, or residual graft material [23]. These findings suggest that the effects of HA may be more pronounced during the early stages of healing, accelerating the initial phases of bone regeneration without necessarily resulting in significant long-term differences.

The observed outcomes in alveolar ridge preservation, ridge augmentation, and maxillary sinus elevation may be explained by biological mechanisms associated with HA.

At the cellular level, this polysaccharide plays an essential role in the extracellular matrix and is involved in key processes of tissue regeneration, particularly bone regeneration. HA exerts its effects through interaction with transmembrane receptors, especially CD44. Binding of HA to this receptor activates intracellular signaling pathways that regulate cell adhesion, proliferation, migration, and differentiation, particularly of mesenchymal cells with osteogenic potential [15,16,17,19,20].

These pathways promote, on one hand, the expression of osteogenic factors such as BMP-2, contributing to osteoblast differentiation and bone formation and mineralization [15,19]. On the other hand, they stimulate angiogenic factors such as VEGF [21], acting on endothelial cells and promoting neovascularization. This improves oxygen and nutrient supply to regenerating tissues, creating a favorable environment for osteoblastic activity and consequently bone formation and remodeling.

In addition, HA exhibits anti-inflammatory and bacteriostatic properties, contributing to a more favorable biological environment for tissue regeneration [15,17,18,19]. The findings of the present review are consistent with those reported in a recent systematic review on fracture healing, which suggested that hyaluronic acid may accelerate bone repair by promoting angiogenesis, enhancing osteoblast activity, and regulating inflammatory responses. Although fracture healing and oral bone regeneration represent different clinical scenarios, both processes share common biological pathways that may explain the beneficial effects observed with HA application [13].

The studies included in this review suggest that HA acts as a bioactive agent promoting a favorable microenvironment for osteogenesis rather than exerting a direct osteoinductive effect [20]. However, these results were not consistent across all studies and were influenced by factors such as graft material type, HA formulation, and healing time.

Although the available clinical evidence suggests that HA may positively influence outcomes such as bone density, new bone formation, and volumetric preservation, the biological mechanisms underlying these effects remain insufficiently understood. The studies included in this review were primarily designed to evaluate clinical, radiographic, histological, and histomorphometric outcomes, whereas direct investigation of the molecular and cellular pathways involved in bone regeneration was generally beyond their scope. Consequently, the proposed effects of HA on angiogenesis, inflammatory modulation, extracellular matrix organization, and osteogenic differentiation remain largely based on experimental evidence and cannot be conclusively confirmed in clinical settings. Future research should therefore move beyond the assessment of clinical outcomes alone and incorporate molecular and cellular analyses to clarify the mechanisms of action of different HA formulations, identify the biological pathways involved, and determine under which clinical conditions HA may provide meaningful regenerative benefits.

Regarding methodology, substantial variability was observed across studies, including graft biomaterials combined with HA, HA formulation, follow-up duration, and outcome assessment tools. The included studies used different biomaterials in association with HA, namely autogenous grafts [20], allogeneic grafts [16,21], xenografts [15,17,18,22], and synthetic materials [19]. Most studies used high-molecular-weight cross-linked HA such as Hyadent BG^®^ [17,19,20,21,22,23] or Perfectha^®^ [15], while others used non-cross-linked HA such as Hyalubrix^®^ [18] or HA matrices [24]. Additionally, one study [16] used HA incorporated directly into the graft biomaterial as an HA-enriched bone substitute, although without detailed specification of its physicochemical characteristics, such as degree of cross-linking.

However, HA molecular weight may influence bone formation. High-molecular-weight HA appears to favour osteogenesis through stimulation of angiogenesis and mesenchymal cell differentiation [21].

Follow-up periods ranged from 2 to 12 months, with 4-month evaluations being the most common and corresponding to a critical phase of bone healing comparable to autogenous graft healing [21]. Some studies reported longer periods between 6 and 8 months, particularly with xenografts [22], while 12-month follow-up allowed assessment of medium-term stability.

Furthermore, considerable variability was observed in outcome assessment methods, including clinical [15,19,20,21,22], radiographic [15,16,17,18,19,20,21,23,24], histomorphometric [15,20,21,22,23,24], and histological [15,17,19,20,21] analyses, which limits direct comparison and clinical extrapolation.

The interpretation of the findings should consider the methodological heterogeneity among the included studies, particularly regarding HA formulations, grafting materials, outcome assessment methods, and follow-up periods. These factors may partly explain the variability observed across studies.

The findings of the present review should also be interpreted in the context of previously published systematic reviews. Lorenzi et al. conducted a systematic review and meta-analysis to evaluate the adjunctive use of hyaluronic acid in bone regeneration procedures. Although the included studies reported encouraging findings, the meta-analysis, based on only three randomized clinical trials, did not demonstrate statistically significant differences in new bone formation or residual graft particles and highlighted the limited amount of available clinical evidence [25]. More recently, Pizzolante et al. systematically reviewed the use of hyaluronic acid in post-extraction socket preservation and concluded that hyaluronic acid may improve alveolar ridge preservation, although further high-quality clinical studies are required [11]. Similarly, Ronsivalle et al. reported the potential of hyaluronic acid to enhance regenerative outcomes in alveolar ridge preservation, while also emphasizing the need for larger, well-designed randomized clinical trials [26]. The present review builds on these previous evidence syntheses by incorporating more recently published clinical studies and by evaluating the adjunctive use of hyaluronic acid across alveolar ridge preservation, ridge augmentation, and maxillary sinus elevation procedures.

From a clinical perspective, it is important to question whether the observed benefits, although statistically significant in some studies, translate into clinically meaningful advantages such as reduced healing time or improved predictability of regenerative procedures. Based on the current evidence, it cannot yet be stated that HA significantly alters clinical decision-making. Therefore, its use should be considered as an adjunct rather than a substitute for conventional bone regeneration protocols.

All things considered, the available evidence suggests that HA may positively influence bone regeneration, particularly in alveolar ridge preservation and ridge augmentation procedures. However, the observed benefits should be interpreted with caution due to the heterogeneity of the available studies.

### Strengths and Limitations of the Present Review

The present systematic review has several strengths. First, it was conducted according to PRISMA 2020 recommendations, and the protocol was registered in PROSPERO, increasing methodological transparency and reducing the risk of reporting bias. Second, only clinical human studies with a control group were included, thereby enhancing the clinical relevance of the findings. Third, multiple outcome measures were evaluated, including clinical, radiographic, histological, and histomorphometric parameters, providing a comprehensive assessment of the potential effects of hyaluronic acid on bone regeneration.

Nevertheless, some limitations should be acknowledged. The number of eligible studies was relatively small, and most studies included limited sample sizes. Considerable heterogeneity was observed regarding surgical procedures, grafting materials, hyaluronic acid formulations, concentrations, molecular weights, application protocols, and follow-up periods. Such variability limits direct comparisons between studies and precluded quantitative meta-analysis. Furthermore, several studies presented methodological concerns related to randomization procedures, allocation concealment, and blinding. Finally, publication bias cannot be excluded, as studies reporting positive outcomes are more likely to be published. A formal assessment of the certainty of evidence using the GRADE approach was not performed. Therefore, although the available evidence suggests beneficial effects of hyaluronic acid as an adjunct in bone regeneration, the overall certainty of the evidence cannot be formally established, and the findings should be interpreted with caution.

## 5. Conclusions

The available evidence suggests that hyaluronic acid (HA) may positively influence bone regeneration when used as an adjunct to graft procedures, particularly in alveolar ridge preservation and ridge augmentation. The main benefits reported include increased new bone formation, improved bone density, and enhanced bone maturation. The current evidence does not support the superiority of any specific HA formulation, and the results remain inconsistent for maxillary sinus augmentation procedures. However, included studies were primarily clinical in nature and do not provide adequate mechanistic evidence to confirm the proposed biological pathways. Given the heterogeneity of the included studies and their methodological limitations, definitive conclusions regarding the clinical effectiveness of HA cannot yet be established. Further well-designed randomized controlled trials with standardized protocols and longer follow-up periods are required to clarify its role in bone regeneration and support evidence-based clinical recommendations.

## Figures and Tables

**Table 1:** PICO Strategy.

**P (Population)**	Adult patients undergoing alveolar ridge preservation, ridge augmentation, or maxillary sinus elevation procedures
**I (Intervention)**	Local application of hyaluronic acid (HA), in any formulation (gel, cross-linked, combined with bone grafts or biomaterials), applied locally during the regenerative procedure
**C (Comparison)**	Bone regeneration procedures performed without HA using conventional biomaterials
**O (Outcomes)**	- Gain in alveolar bone volume, width, and height - Percentage of newly formed bone - Bone density - Bone maturation

**Table 2:** Inclusion criteria.

Inclusion Criteria
Articles published in English, Portuguese, or Spanish.
Articles published between January 2016 and January 2026.
Studies conducted in adults aged ≥ 18 years.
Clinical studies evaluating alveolar ridge preservation, post-extraction bone regeneration, or maxillary sinus elevation procedures reporting relevant clinical, radiographic, histological, or histomorphometric quantitative outcomes.
Studies investigating the local application of hyaluronic acid in any formulation.

**Table 3:** Results Obtained from the Search Strategy.

Database	Search Strategy	Articles Identified
PubMed	(“Hyaluronic acid”[MeSH Terms]) AND (“oral surgical procedures”[MeSH Terms])	45
Science Direct	“Hyaluronic acid” AND “maxillary sinus”	2
Google Scholar	“Hyaluronic acid” “bone regeneration” “tooth extraction”-animals-reviews	607
Wiley Online Library	“Hyaluronic acid” “bone regeneration” “maxillary sinus”	74

**Table 4:** Characteristics of Included Studies: Study Design and Hyaluronic Acid (HA) Formulation.

Study	Study Design	Commercial Product	Cross-Linked HA	Molecular Weight	Concentration
Abaza et al. (2024) [15]	RCT	Perfectha^®^	Yes	NR	20 mg/mL
Kloss et al. (2024) [16]	Comparative clinical study	Maxgraft^®^ + Hya	HA-enriched allograft	NR	NR
Husseini et al. (2023) [17]	Split-mouth RCT	Hyadent BG^®^	Yes	High MW	16 mg/mL
Abdelzaher et al. (2022) [18]	RCT	Hyalubrix^®^	No	High MW (1.5–2.0 MDa)	15 mg/mL
Baiomy et al. (2020) [19]	RCT	Hyadent^®^	Yes	High MW	16 mg/mL
Taman et al. (2017) [20]	Split-mouth RCT	Hyadent^®^	Yes	High MW	16 mg/mL
Helal et al. (2025) [21]	Split-mouth RCT	Hyadent BG^®^	Yes	High MW	16 mg/mL
Kauffmann et al. (2023) [22]	RCT	Hyadent BG^®^	Yes	High MW	16 mg/mL
Velasco-Ortega et al. (2020) [23]	RCT	Hyadent BG^®^	Yes	High MW	16 mg/mL
Dogan et al. (2017) [24]	Split-mouth RCT	HA matrix (ester)	Yes	NR	20–60 mg/mL

Abbreviations: HA, hyaluronic acid; MW, molecular weight; NR, not reported.

**Table 5:** Risk of Bias Assessment of Included Studies by Assessment Tool.

Authors (Year)	Study Type	Assessment Tool	Overall Risk of Bias
Abaza et al. (2024) [15]	RCT	RoB 2	Some concerns
Kloss et al. (2024) [16]	Comparative clinical study	ROBINS-I	Moderate
Husseini et al. (2023) [17]	Split-mouth, double-blind RCT	RoB 2	Some concerns
Abdelzaher et al. (2022) [18]	RCT	RoB 2	Some concerns
Baiomy et al. (2020) [19]	RCT	RoB 2	Some concerns
Taman et al. (2017) [20]	Split-mouth RCT	RoB 2	Some concerns
Helal et al. (2025) [21]	Split-mouth, double-blind RCT	RoB 2	Low risk
Kauffmann et al. (2023) [22]	RCT	RoB 2	Some concerns
Velasco-ortega et al. (2020) [23]	RCT	RoB 2	Low risk
Dogan et al. (2017) [24]	Split-mouth RCT	RoB 2	Low risk

**Table 6:** Characteristics and Main Findings of the Included Studies.

Study	Design	Clinical Indication	Sample Size	Intervention Group	Control Group	Follow-Up	Outcome Measures	Main Findings
Abaza et al. (2024) [15]	RCT	Alveolar ridge preservation	36 patients (36 sockets)	Xenograft + HA	Xenograft alone	12 months	Bone width, bone loss, new bone formation, mature bone, histology	Increased new and mature bone formation, reduced bone loss, and improved trabecular architecture.
Kloss et al. (2024) [16]	Comparative clinical study	Alveolar ridge preservation	40 patients	Allograft + HA	Allograft alone	12 months	Vertical and horizontal bone loss, volume loss, bone density	Reduced vertical bone loss, graft shrinkage, and volume loss; increased bone density.
Husseini et al. (2023) [17]	Split-mouth RCT	Alveolar ridge preservation	7 patients (14 sockets)	DBBM + HA	DBBM alone	4 months	Linear and volumetric bone resorption, histology	Reduced alveolar ridge resorption and improved bone healing.
Abdelzaher et al. (2022) [18]	RCT	Alveolar ridge preservation	12 patients (20 sockets)	Xenograft + HA + PRF	Xenograft + PRF	3 months	Bone density	Significantly increased bone density.
Baiomy et al. (2020) [19]	RCT	Alveolar ridge preservation	30 patients	Osteon II Collagen + HA	Sticky bone graft	6 months	Bone dimensions, bone volume, bone density, histology	Improved bone height, volume, density, and maturation.
Taman et al. (2017) [20]	Split-mouth RCT	Alveolar ridge preservation	10 patients (20 sockets)	Autogenous graft + HA	Autogenous graft alone	2 months	Bone density, histology	Increased bone density and osteoblastic activity.
Helal et al. (2025) [21]	Split-mouth RCT	Ridge augmentation	10 patients (20 sites)	CAD-CAM block + HA	CAD-CAM block alone	12 months	Bone gain, bone loss, new bone formation, VEGF expression	Enhanced bone regeneration, angiogenesis, and graft integration.
Kauffmann et al. (2023) [22]	RCT	Ridge augmentation	11 patients (27 sites)	DBBM + HA	DBBM alone	6 months	New bone formation, mineralized bone, residual biomaterial	Increased new bone formation and reduced residual biomaterial.
Velasco-Ortega et al. (2020) [23]	RCT	Maxillary sinus elevation	24 patients	TCP + HA	TCP alone	9 months	New bone formation, bone gain, residual biomaterial	No significant benefit compared with control.
Dogan et al. (2017) [24]	Split-mouth RCT	Maxillary sinus elevation	13 patients (26 sinus elevations)	CHBG + HA matrix	CHBG alone	4 months	New bone formation (micro-CT and histomorphometry)	Significantly increased new bone formation.

Abbreviations: HA, hyaluronic acid; RCT, randomized controlled trial; DBBM, deproteinized bovine bone mineral; PRF, platelet-rich fibrin; CAD-CAM, computer-aided design/computer-aided manufacturing; TCP, β-tricalcium phosphate; CHBG, cancellous human bone graft; VEGF, vascular endothelial growth factor; micro-CT, micro-computed tomography.

## Data Availability

No new data were created or analyzed in this study. All data supporting the findings of this study are contained within the published articles included in the review and are cited in the reference list.

## Supplementary Materials

The following supporting information can be downloaded at: https://www.mdpi.com/article/10.3390/biomedicines14071514/s1, Table S1. RoB 2 Assesment for RCT. Table S2. ROBINS-I Assesment for Non RCT. Figure S1. RoB 2 Assesment for RCT- Traffic-Light Plot. Figure S2. ROBBINS-I Assesment for Non RCT- Traffic-Light Plot. References [15,16,17,18,19,20,21,22,23,24] are cited in the supplementary materials

## Abbreviations

The following abbreviations are used in this manuscript:

ABBM	Anorganic bovine bone matrix
HA	Hyaluronic acid
CAD-CAM	Computer-aided design/computer-aided manufacturing
CBCT	Cone-beam computed tomography
CGF	Concentrated growth factors
CHBG	Collagenated heterologous bone graft
DBBM	Demineralized bovine bone material
HU	Hounsfield unit
I-PRF	Injectable platelet-rich fibrin
PTFE membrane	Polytetrafluoroethylene membrane
mm	Millimeters
PRF	Platelet-rich fibrin
RCT	Randomized controlled trial
TCP	Tricalcium phosphate
xHyA	Cross-linked high-molecular-weight hyaluronic acid
