# Supplementary material for: Hyaluronic Acid as an Adjunct in Bone Regeneration—A Systematic Review
Source: Biomedicines. 2026 Jul 5;14(7):1514. doi: 10.3390/biomedicines14071514 (PMC13406002; doi:10.3390/biomedicines14071514)

Supplementary Materials- Detailed domain-specific assessments

Table S1-RoB 2 Assesment for RCT

Study	Randomization Process	Deviations from Intended Interventions	Missing Outcome Data	Measurement of Outcome	Selection of Reported Results	Overall
**Abaza et al. (2024) [15]**	Low	Some concerns	Low	Low	Some concerns	Some concerns
**Husseini et al. (2023) [17]**	Low	Low	Low	Low	Some concerns	Some concerns
**Abdelzaher et al. (2022) [18]**	Some concerns	Low	Low	Low	Some concerns	Some concerns
**Baiomy et al. (2020) [19]**	Some concerns	Low	Low	Low	Some concerns	Some concerns
**Taman et al. (2017) [20]**	Some concerns	Low	Low	Low	Some concerns	Some concerns
**Helal et al. (2025) [21]**	Low	Low	Low	Low	Low	Low risk
**Kauffmann et al. (2023) [22]**	Low	Some concerns	Low	Low	Some concerns	Some concerns
**Velasco-Ortega et al. (2020) [23]**	Low	Low	Low	Low	Low	Low risk
**Dogan et al. (2017) [24]**	Low	Low	Low	Low	Low	Low risk

Table S2-ROBINS-I Assesment for Non RCT

Study	Confounding	Selection of Participants	Classification of Interventions	Deviations from Intended Interventions	Missing Data	Measurement of Outcomes	Selection of Reported Results	Overall
**Kloss et al. (2024) [16]**	Moderate	Moderate	Low	Low	Low	Low	Low	Moderate

Figure S1-RoB 2 Assesment for RCT -Traffic-Light Plot

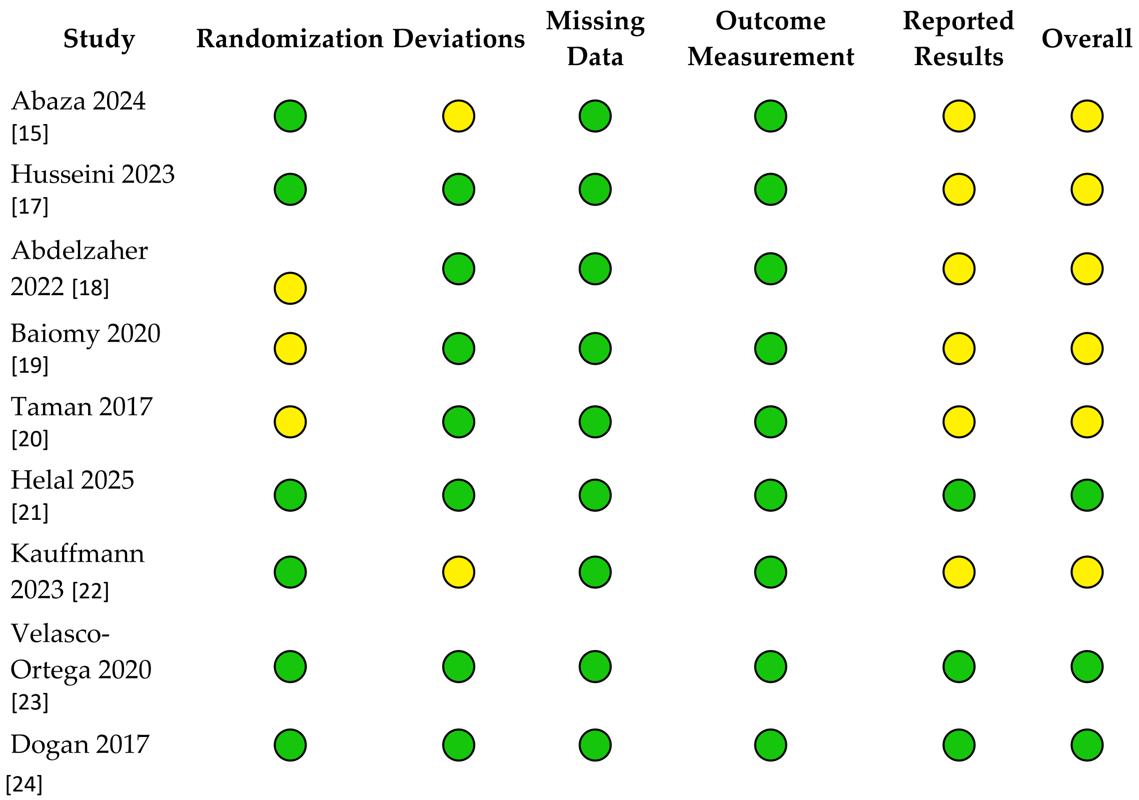

Figure S2. ROBBINS-I Assesment for Non RCT- Traffic-Light Plot

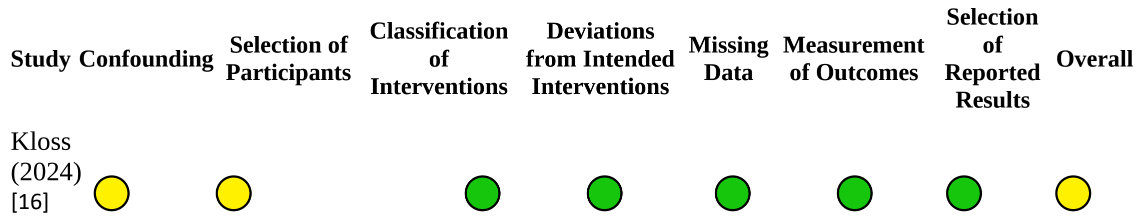

Supplement: Supplementary file 1 [file biomedicines-14-01514-s001.zip › biomedicines-4358239-supplementary.pdf]
